# Characterization of the Oral Fungal Microbiome (Mycobiome) in Healthy Individuals

**DOI:** 10.1371/journal.ppat.1000713

**Published:** 2010-01-08

**Authors:** Mahmoud A. Ghannoum, Richard J. Jurevic, Pranab K. Mukherjee, Fan Cui, Masoumeh Sikaroodi, Ammar Naqvi, Patrick M. Gillevet

**Affiliations:** 1 Center for Medical Microbiology, Department of Dermatology, School of Medicine, Case Western Reserve University and University Hospitals Case Medical Center, Cleveland, Ohio, United States of America; 2 Department of Biological Sciences, School of Dental Medicine, Case Western Reserve University, Cleveland, Ohio, United States of America; 3 Microbiome Analysis Center, Department of Environmental Science and Policy, George Mason University, Fairfax, Virginia, United States of America; University of Birmingham, United Kingdom

## Abstract

The oral microbiome–organisms residing in the oral cavity and their collective genome–are critical components of health and disease. The fungal component of the oral microbiota has not been characterized. In this study, we used a novel multitag pyrosequencing approach to characterize fungi present in the oral cavity of 20 healthy individuals, using the pan-fungal internal transcribed spacer (ITS) primers. Our results revealed the “basal” oral mycobiome profile of the enrolled individuals, and showed that across all the samples studied, the oral cavity contained 74 culturable and 11 non-culturable fungal genera. Among these genera, 39 were present in only one person, 16 genera were present in two participants, and 5 genera were present in three people, while 15 genera (including non-culturable organisms) were present in ≥4 (20%) participants. *Candida* species were the most frequent (isolated from 75% of participants), followed by *Cladosporium* (65%), *Aureobasidium*, Saccharomycetales (50% for both), *Aspergillus* (35%), *Fusarium* (30%), and *Cryptococcus* (20%). Four of these predominant genera are known to be pathogenic in humans. The low-abundance genera may represent environmental fungi present in the oral cavity and could simply be spores inhaled from the air or material ingested with food. Among the culturable genera, 61 were represented by one species each, while 13 genera comprised between 2 and 6 different species; the total number of species identified were 101. The number of species in the oral cavity of each individual ranged between 9 and 23. Principal component (PCO) analysis of the obtained data set followed by sample clustering and UniFrac analysis revealed that White males and Asian males clustered differently from each other, whereas both Asian and White females clustered together. This is the first study that identified the “basal mycobiome” of healthy individuals, and provides the basis for a detailed characterization of the oral mycobiome in health and disease.

## Author Summary

We characterized the fungal microbiome (mycobiome) of the oral cavity in healthy individuals. Our results demonstrate that the fungal component of the oral microbiome is diverse as revealed by the presence of 74 culturable and 11 non-culturable fungal genera in the oral cavity. A total of 101 species were identified, with between 9 and 23 culturable species present in each person. Fifteen genera (which included four known pathogenic fungi and non-culturable organisms) were present in ≥20% of the tested samples; *Candida* species were the most frequently obtained genera, isolated from 75% of all study participants, followed by *Cladosporium* (65%), *Aureobasidium*, Saccharomycetales (50% for both), *Aspergillus* (35%), *Fusarium* (30%), and *Cryptococcus* (20%). The remaining fungi detected in the oral wash samples represent organisms likely originating from the environment. This is the first study that identified the “basal mycobiome” of healthy individuals, and provides the basis for a detailed characterization of the oral mycobiome in health and disease.

## Introduction

Organisms residing in the oral cavity and their collective genome–the oral microbiome–are critical components of health and disease. Disruption of the oral microbiome has been proposed to indicate, trigger, or influence the course of oral diseases, especially among immunocompromised patients (e.g. HIV-infected or cancer patients) [1]–[3]. Although fungi, particularly *Candida*, are important components of oral microbiota and are influenced by the immune status and therapy of affected individuals, studies of oral microbiota have focused largely on the bacterial components. In the only oral microbiome study to date that included some fungal profiling, Aas *et al.* [4] reported the presence of *Candida albicans* and *Saccharomyces cerevisiae* in the subgingival plaque microbiota of HIV-infected patients. These investigators employed a PCR-based approach using the 18S rDNA primers (that amplify *Candida* spp. and eight divergent fungal genera only) to characterize the fungi present in the plaques. The approach used by this group provided only a limited snap shot of the fungal members of the microbial biome.

To obtain a more comprehensive profile of the fungal microbiome (mycobiome), in this study we utilized a novel Multitag Pyrosequencing (MTPS) approach to interrogate the fungal taxa in the oral cavity using universal internal transcribed spacer (ITS) primers, which have broad fungal specificity [5]–[13]. Using this approach, we characterized the “basal” mycobiome profile of 20 healthy individuals, and showed that across all the samples studied, the oral cavity contained 74 culturable and 11 non-culturable fungal genera. Among these culturable genera, 61 were represented by one species each, while 13 genera comprised between 2 to 6 different species; the total number of species identified were 101. This is the first study that identified the “basal mycobiome” of healthy individuals, which provides the basis for detailed characterization of the oral mycobiome in health and disease.

## Materials and Methods

### Ethics Statement

Written informed consent was obtained from all participants in this study. Recruitment of study participants was performed according to protocol (number 20070413) approved by the Human Subjects Institutional Review Board (IRB) of Case Western Reserve University, Cleveland, Ohio.

### Study Participants

Oral rinse samples were obtained from 20 healthy individuals after informed consent and following review of the IRB at Case Western Reserve University/University Hospitals Case Medical Center. The individuals were all from the Cleveland area and on standard Western diets. Summary demographic information of the study participants is provided in Table 1. Self-reported ethnicities of study participants were classified based on the US Census criteria for classification of race (http://www.census.gov), in which race has been classified as White (including Hispanic, East Indian, or European), Black/African-American, Asian, Native American/Native Alaskan etc. Inclusion criteria were: >18 years of age, non-smoking, no recent antifungal use, and no clinical signs of oral mucosal disease. Exclusion criteria were: (1) a history of receiving medication or treatment with topical or systemic steroids, pregnancy, and (2) insulin-dependent diabetes mellitus (IDDM).

**Table 1:** Summary of demographic characteristics and sequencing information for study participants.

Sample ID	Sample Collection Date	Age	Gender	Ethnicity
A1	16-May	43	F	Asian
A2	16-May	55	M	White
A3	16-May	35	M	Asian
B1	16-May	21	F	Asian
B2	16-May	56	M	White
B3	16-May	39	F	Asian
C1	16-May	22	F	White
C2	16-May	31	F	Asian
C3	16-May	35	M	Asian
D1	16-May	60	M	Asian
D2	16-May	50	M	Asian
E1	21-May	44	M	Asian
E2	21-May	25	F	African-American
E3	21-May	24	F	White
F1	21-May	24	F	African-American
F2	21-May	26	M	White
G1	21-May	56	M	White
G2	21-May	35	M	Asian
G3	21-May	55	M	White
H2	21-May	26	M	White

### Collection of Oral Rinse

Concentrated oral rinse has been previously used to detect the presence of oral bacteria and fungi [14]–[16]. We selected oral rinse for our studies because: (a) it is relatively simple and noninvasive to collect, (b) safer to handle than other body fluids (e.g. serum), (c) the oral cavity is the major entry point of microbes into the body and (d) oral rinse enables the collection of organisms from the dorsum of the tongue and the oral mucosal environment. Oral samples were collected at least 1 h after a meal, at approximately the same time (9–11 AM), to avoid contamination of samples with extraneous components and standardize the possible impact of variation in salivary flow rates. Study participants rinsed their mouth (swish/gargle) with 15 mL sterile phosphate buffered saline (PBS) for 1 min, and expectorated the contents of the mouth into a 50 mL centrifuge tube. The collected samples were centrifuged at 4000 rpm for 20 min at 4°C to separate the cells (pellet) from extracellular soluble components (supernatant). The cell pellet was used for DNA extraction or stored at −80°C until the time of analysis.

### DNA Extraction and PCR Analysis

The first step in the mycobiome analysis was extraction of DNA from the cell pellet (obtained from oral rinse samples, above) followed by PCR analysis. Samples were extracted individually using the Fast DNA Spin Kit for fungi following manufacturer's instructions (BIO 101; Vista, CA). Each extraction tube was agitated three times using a Fast Prep FP120 instrument at a speed setting of 5 for 30 s. Tubes were cooled on ice between agitations. The ITS1 region from DNA sample extracts was amplified in triplicate using primers with high specificity for ascomycete fungi (fluorescently-labeled forward primer ITS1F (CTTGGTCATTTAGAGGAAGTAA) and unlabeled reverse primer ITS2 (GCTGCGTTCTTCATCGATGC). The ITS primers were selected in this study to detect the presence of various fungi since these primers are able to detect consensus sequences present in a broad range of fungal organisms, and have been used for the detection of yeasts (including *Candida* spp.), moulds and dermatophytes [5]–[13]. The reactions were carried out on ∼10 ng template DNA, in 20-µl (final volume) reaction mixtures consisting of 1×PCR buffer, 0.01% bovine serum albumin, 2.5 mM MgCl_2_, each dNTP at a concentration of 0.25 mM, each primer at a concentration of 0.5 µM, and 0.5 U of AmpliTaq Gold DNA polymerase (ABI, Foster City, CA). Initial denaturation at 94°C for 11 min was followed by 35 cycles of denaturation for 30s each at 94°C, annealing at 50°C for 30 s, and progressive extension at 72°C for 2 min. Following the 35 cycles there was a final extension time of 30 min to minimize artifacts induced by TAQ polymerase. Fungal PCR products were separated on the SCE 9610 capillary DNA sequencer (Spectrumedix LLC, State College, PA) using GenoSpectrum software to convert fluorescent output into electropherograms. Relative peak abundance of fungal amplicons was calculated by dividing individual peak heights by the total peak heights in a given electropherogram using a custom PERL script. Interleaved, normalized abundances were compared as stacked histograms using Microsoft Excel. Mean normalized abundance for each amplicon was calculated from the three PCR replicates of each sample, excluding means below 1%. Results were analyzed by visual inspection and Principal Coordinate (PCO) analysis using Multivariate Statistical Package (MVSP, Kovach Computing Services, Wales, UK). Normalized abundance of each peak in the electropherogram was calculated with respect to the total peak area, since it is not possible to calculate absolute abundances with either the LH-PCR or MTPS technology.

### Mycobiome Analysis

Mycobiome analysis was performed using multitag 454 pyrosequencing (MTPS) technique, which can be used for detailed characterization of nucleic acids and has the advantages of accuracy, flexibility, parallel processing, and easy automation potential [17]. In this technique, organism-specific sequences are amplified, and the PCR amplicons are converted to single-stranded DNA templates and immobilized onto streptavidin-coated beads. Next, an enzymatic cascade facilitates the simultaneous synthesis of complementary DNA. As each nucleotide is incorporated into the newly synthesized strand, a luciferase-dependent bioluminescent signal is generated, with the intensity of each signal being proportional to the number of incorporated nucleotides. The bioluminescent signal is detected and analyzed by the instrument in real time, with the resulting generation of a pyrogram that consists of a series of peaks whose temporal relationship and height reflect the DNA sequence. Specifically, we generated a set of 24 emulsion PCR fusion primers that contain the 454 emulsion PCR adapter, joined to a 7 base “barcode” along with the appropriate target primers. Specifically for this experiment, we used the A Adapter with a barcode and the ITS1F sequence for the forward primer and the B Adapter with the ITS4A sequence (i.e. without a tag) for the reverse primer. All sequences were read from the A Adapter side. Thus, each oral rinse sample was amplified with a uniquely barcoded set of forward and reverse rRNA primers and then up to 24 samples were pooled and subjected to emulsion PCR and pyrosequenced using a GS-FLX pyrosequencer. Data from each pooled sample were “deconvoluted” by sorting the sequences into bins based on the barcodes using custom PERL scripts. Thus, we were able to normalize each sample by the total number of reads from each barcode. Several groups have subsequently employed various barcoding strategies to analyze multiple samples and this strategy is now well accepted [18]–[21].

### Data Analysis

We developed a custom PERL script to demultiplex the MTPS data by sorting the sequences into bins based on the barcodes and the taxa in the samples, automatically blasting the pyrosequence data against Genbank (98% cutoff, which was sufficient for species identification). The annotations for each sequence were downloaded and a PERL script used to tabulate the taxa as a percentage of the total oral community in each sample. Fungal ITS sequences were compared with the Assembling Fungal Tree of Life (AFTOL) database using the BLAST interface of Web Accessible Sequence Analysis for Biological Inference (WASABI) as well as against the NCBI nucleotide database.

Principal Coordinate (PCO) analysis has been recognized as a simple and straight-forward method to group or separate samples in a dataset, and has been used in disease-association studies [22],[23]. In the current study, PCO was used to analyze the MTPS results using the Multivariate Statistical Package, MVSP (Kovach, Wales, UK) and SAS (Cary, NC). The PCO analysis performs an Eigen analysis on the data matrix using a Brays Curtis distance metric. Graphically, PCO is a rotation of a swarm of data points in multidimensional space so that the axis with the greatest variance is the first principal component axis. The second axis orthogonal to the first is the second principal component and represents the second greatest variance of the data. The first two or three principal components generally account for most of the variance of the data.

### UniFrac Analysis

To compare the phylogenetic distribution between the various gender and race classes and confirm the results of PCO analysis, we used UniFrac significance test [24],[25]. This test measures the probability that the designated classes are different based on the phylogenetic relatedness and the abundance of each cluster. We first clustered the MTPS reads using CD-HIT [26] to reduce the number of sequences used in the multiple alignment. These clusters were annotated by the number of reads in each sample and then aligned using KALIGN [27]. A tree file was constructed following the multiple alignment using PAUP and an environment file was defined for each sample using a custom PERL script. These two files were then loaded into the UniFrac online server (http://bmf2.colorado.edu/unifrac/index.psp). The analysis was weighted using the abundance of the sequence in each cluster. The *P*-value generated from the UniFrac significance test describes the degree of similarity between classes. Values reported were Bonferroni corrected for the number of sequences used in comparisons.

## Results

### Participant Demographics

The demographic characteristics of the study participants were: 21–60 years of age, 8 females and 12 males, no history of smoking, insulin-dependent diabetes mellitus, or active medications; the self-reported ethnicities of study participants were White (n = 8; 2 females, 6 males), Asian (Han Chinese, Indian or Bangladeshi) (n = 10; 4 females, 6 males), or African-American (n = 2, both females). All participants were from the metropolitan Cleveland Ohio area, and were Faculty, staff or students at Case Western Reserve University (Table 1). Study participants were assumed to consume a varied “Western” diet. Analysis of complex population genetic structure to control for differences in continental ancestory was not performed for these studies.

### Identification of Different Genera in Oral Rinse Samples

**Figure 1:**
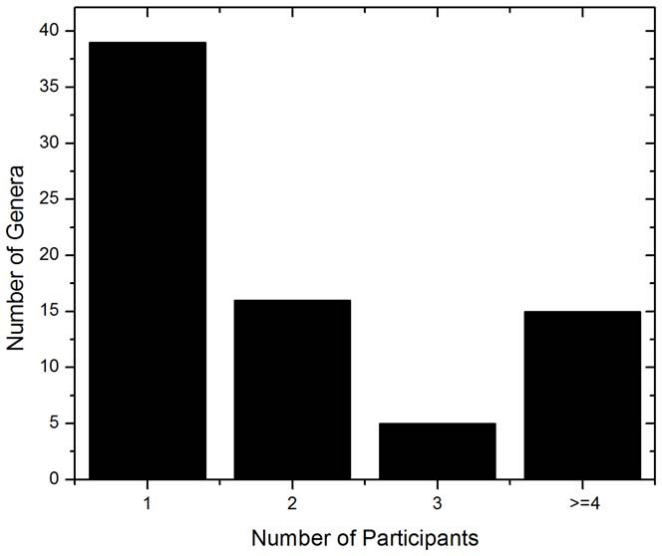
Frequency distribution of identified fungal genera among the study participants.

**Figure 2:**
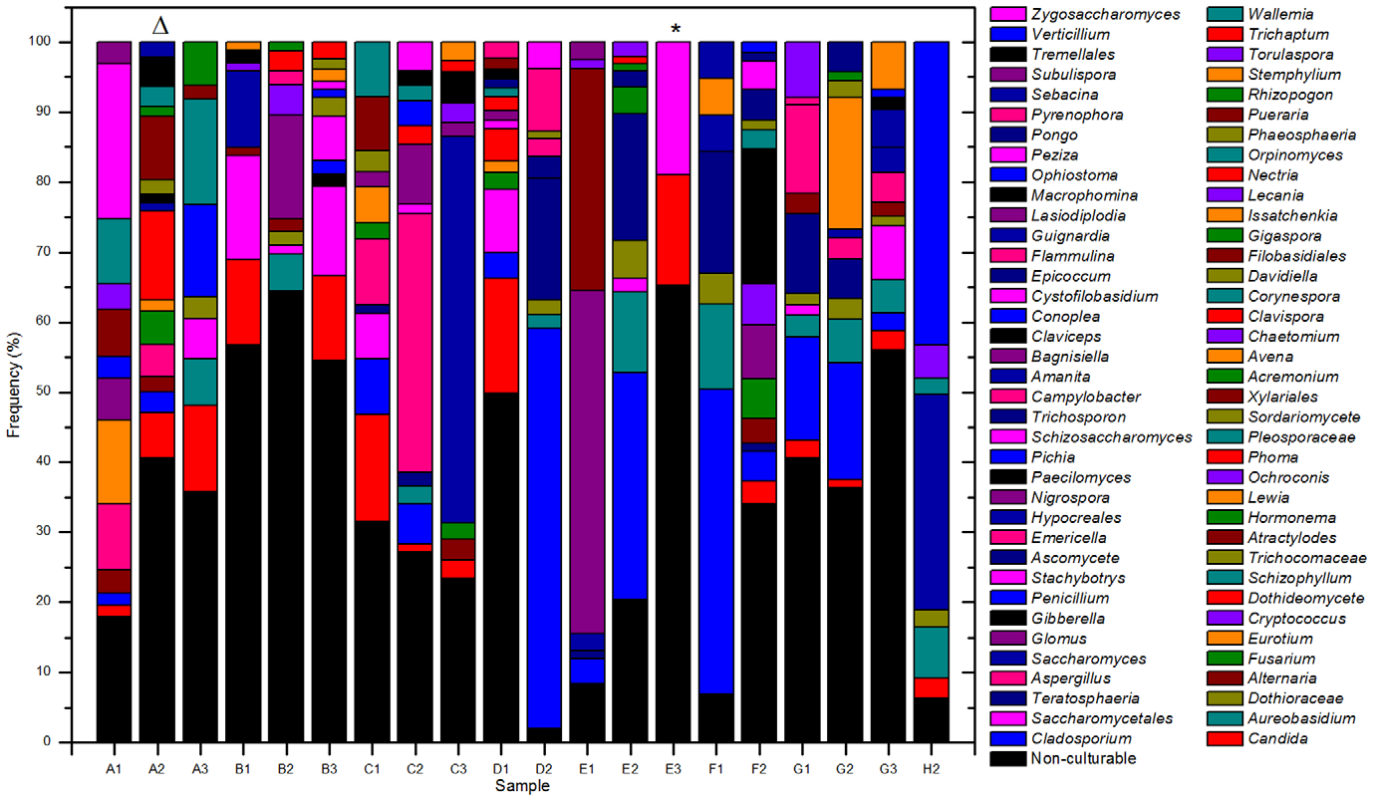
Overall distribution of fungi in oral rinse samples obtained from 20 healthy individuals. The triangle and asterisk indicate samples containing 16 and 3 fungal genera, respectively. See Table 1 for sample details.

The ITS-based sequencing runs produced 39,226 sequence reads of which 36,155 contained identifiable tags. Of those, 34,049 sequences (94%) were longer than 100 bases and were used in the analysis. Our analyses revealed an average of 1,702 sequences per sample with an average length of 248 bases (Table 2). A local copy of Genbank was searched using megablast and the highest hit (98% cutoff) was compiled using the score for each sequence and the results were then tabulated using a custom PERL script (see Supplemental Table S1 for sequence details). Figure 1 shows the genera identified in the oral rinse samples collected from the 20 participants that were ≥1% of the community in individual samples. Across all the samples studied, the oral cavity contained 74 culturable and 11 non-culturable fungal genera (supplemental Tables S2 and S3). Thirty-nine genera were present in only one person, 16 genera were present in two participants, 5 genera were present in three people, while 15 genera (including the non-culturable genera) were present in 4 or more people (Fig. 1). Among the culturable genera, 61 were represented by one species each, while 13 genera comprised between 2 to 6 different species; the total number of species identified were 101. The number of species in the oral cavity of each individual ranged between 9 and 23 (supplemental Tables S2 and S3). More than 10 different genera with an abundance of >1% were detected in 70% (14/20) of the samples analyzed (supplemental Tables S2 and S3). When compared across all 20 individuals, four genera were present in the oral rinse of 10 or more study participants: *Candida* (15/20), *Cladosporium* (13/20), *Aureobasidium* (10/20), and organisms belonging to the family Saccharomycetales (10/20). Interestingly, a large percentage (36.1%) of fungi belonged to non-culturable category. The minimum number of genera identified in a sample (sample E3) was 3 (*Candida*, 15%; Saccharomycetales, 18.1%; unculturable, 62.3%), while the maximum number of genera identified was 16 in sample A2, which included *Candida* (5.5%), Dothediomycete (10.7%), *Fusarium* (4.1%), *Aspergillus* (3.9%), and Xylariales (7.7%) (Fig. 2). The non-culturable genera/family detected in the oral samples included *Glomus*, Leptosphaeriaceae, Ascomycete, Basidiomycete, Ectomycorrhiza, Endophytic fungi, and Glomeromycete.

**Table 2:** Summary of pyrosequencing analysis.

Sample ID	Number of Reads	Average Read Length
A1	1819	246
A2	2546	263
A3	1961	231
B1	3688	251
B2	2528	254
B3	815	254
C1	1392	256
C2	2739	271
C3	1202	269
D1	2185	259
D2	1359	217
E1	1890	218
E2	559	265
E3	1595	254
F1	1249	211
F2	2357	243
G1	615	257
G2	1339	252
G3	976	241
H2	1228	247
**Average**	**1702**	**248**

### Distribution of Fungi in the Oral Cavity of Healthy Individuals

**Figure 3:**
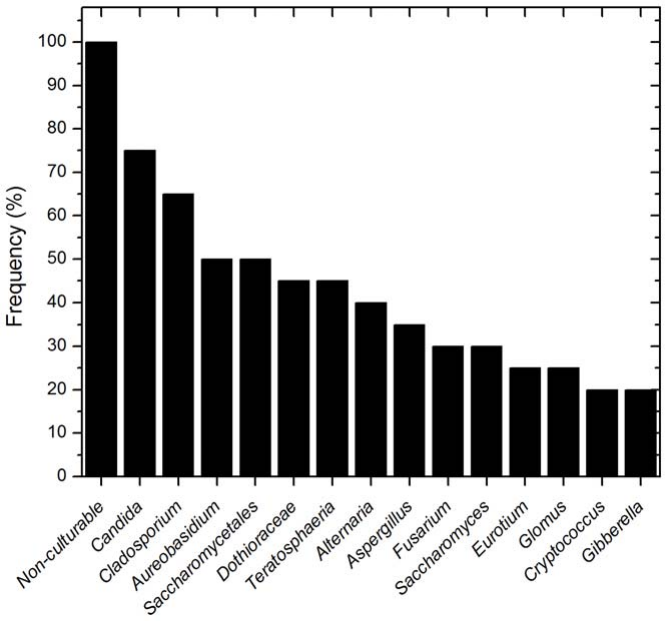
Frequency of fungal genera present in more than 20 percent of the tested samples.

To determine the basal fungal distribution in healthy individuals, we identified fungi that were present in at least 20% (4/20 individuals sampled) of the study participants. This analysis revealed that 15 genera were present in ≥20% of the tested samples (including non-culturable fungi, Fig. 3). Among these samples, *Candida* species were the most frequently obtained genera, isolated from 75% of all study participants, followed by *Cladosporium* (65%), *Aureobasidium* and Saccharomycetales (50% for both). Other fungi that were present in the oral cavity of healthy individuals were *Aspergillus* (35%), *Fusarium* (30%), and *Cryptococcus* (20%). Fifty three percent (39/74) of the identified genera were observed only once in the tested samples.

Analysis of the species distribution of the oral mycobiome revealed that 12 fungi were represented by two or more species in the oral rinse samples (Supplemental Tables S2 and S3). The highest number of species was detected for *Aspergillus* (6 species), followed by *Candida* (5 species), *Cladosporium* (4 species), *Fusarium* (3 species), and *Penicillium* (3 species) (Table 3). *Candida albicans* was identified in 40% of the participants (8/20), while the non-*albicans Candida* species indentified were: *C. parapsilosis* (15%), *C. tropicalis* (15%), *C. khmerensis* and *C. metapsilosis* (in 5% of the subjects). Two species each of *Alternaria*, *Cryptococcus*, *Ophiostoma*, *Glomus*, *Phoma*, *Schizosaccharomyces*, and *Zygosaccharmoyces* were identified in the participants.

**Table 3:** Distribution of different fungal species in oral mycobiome of healthy individuals.

Fungal genera	Species	Frequency
*Alternaria*	*tenuissima*	2
	*triticina*	1
*Aspergillus*	*amstelodami*	2
	*caesiellus*	1
	*flavus*	1
	*oryzae*	1
	*penicillioides*	1
	*ruber*	2
*Candida*	*albicans*	8
	*khmerensis*	1
	*metapsilosis*	1
	*parapsilosis*	3
	*tropicalis*	3
*Cladosporium*	*cladosporioides*	10
	*herbarum*	2
	*sphaerospermum*	1
	*tenuissimum*	1
*Cryptococcus*	*cellulolyticus*	1
	*diffluens*	1
*Fusarium*	*culmorum*	2
	*oxysporum*	1
	*Poae*	1
*Glomus*	*fulvum*	1
	*mosseae*	3
*Ophiostoma*	*floccosum*	1
	*pulvinisporum*	1
*Penicillium*	*brevicompactum*	1
	*glabrum*	1
	*spinulosum*	1
*Phoma*	*foveata*	1
	*plurivora*	1
*Saccharomyces*	*bayanus*	2
	*cerevisiae*	6
	*ellipsoideus*	2
*Schizosaccharomyces*	*japonicus*	1
	*pombe*	1
*Zygosaccharomyces*	*pseudorouxii*	1
	*Rouxii*	1

### Changes in Mycobiome are Associated with Gender and Ethnicity

**Figure 4:**
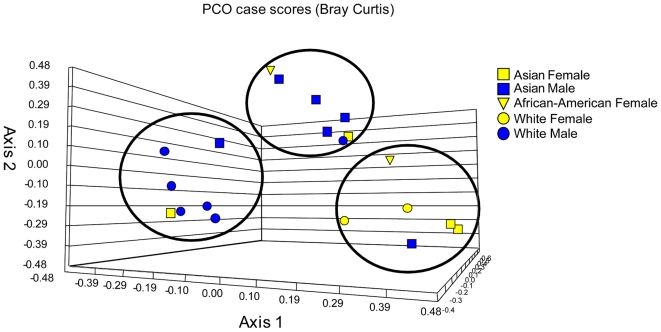
Principal component (PCO) analysis of distribution of fungal genera in oral samples of 20 healthy individuals.

To investigate whether there is an association between any of the subject demographics and changes in mycobiome, we performed PCO analysis and sample clustering followed by UniFrac analysis. Our analysis revealed that White and Asian males clustered differently from each other, whereas both Asian and White females clustered together (Fig. 4). UniFrac analysis of females, White males, and Asian males showed that each of these classes was significantly different from the other (Table 4), supporting the PCO clustering denoted by the circles in Figure 4. These data suggest a trend of association between gender/ethnicity and the oral mycobiome.

**Table 4:** UniFrac analysis of data belonging to clusters of females, White males, and Asian males.

	Asian Males	Females	White Males
Asian Males	0	< = 0.03	< = 0.03
Females	< = 0.03	0	< = 0.03
White Males	< = 0.03	< = 0.03	0

## Discussion

In the current study, we demonstrated the presence of 74 culturable and 11 non-culturable fungal genera in the oral cavity of healthy individuals, with between 9 and 23 culturable species present in each person, representing a total of 101 species for all study participants.

Our results demonstrate that the fungal component of the oral microbiome is not limited to a few species, principally *Candida*; rather it is represented by a large number of diverse fungi. The perception that fungi in the oral cavity are limited to only few species originated from previous studies that relied upon the use of culture-based methods or species-specific targeted PCR approach. In addition to *Candida*, other fungi previously reported in the oral cavity include *S. cerevisiae*, *Penicillium*, *Geotrichum*, *Aspergillus*, *Scopulariopsis*, *Hemispora*, and *Hormodendrum* [28]–[30]. In the current study, we used the pan-fungal ITS probes in conjunction with 454 pyrosequencing, which allowed us to identify oral fungi in a highly specific and sensitive manner. This real-time DNA sequencing method allows rapid analysis of sub-sequences within the ITS regions and comparison with nucleic acid sequence databases, thereby facilitating rapid and accurate species level identification of fungi [31]. Another reason for the successful identification of a large number of fungal species (101 species) in the oral cavity is the ability of MTPS to perform concomitant analysis of multiple samples.

Only two previous studies have investigated the profile of microbes present in the oral cavity of healthy individuals and both focused on the bacterial microbiome [32],[33]. Aas *et al.* [32] used PCR amplification of 16S rRNA genes followed by sequencing to analyze nine oral sites from five clinically healthy subjects, and reported detection of 141 bacterial species across all 5 subjects, of which over 60% were non-culturable. The number of predominant species per individual ranged from 34 to 72. In our study, we found a total of 101 fungal species across all 20 individuals, with the number of species per individual ranging between 9 and 23. Diversity of fungal taxa has also been shown to exist in murine models [34]. Our results suggest that the distribution and profile of fungal species in the oral cavity of healthy individuals is complex, and similar to that of oral bacterial microbiome with respect to the number of species identified.

While the fungal component of the oral cavity has not been investigated in healthy individuals, a previous study identified fungi present in the oral cavity of HIV-infected patients [4]. In this study, Aas *et al.* [4] analyzed sub-gingival plaque of 14 HIV-infected patients, and reported the presence of *S. cerevisiae* in 4 and *C. albicans* in 2 patients. No other fungal species were detected in analysis of 306 18S rDNA clones. In contrast, we found that the oral cavity of healthy individuals had 101 fungal species. The reason for this difference could be attributed to differences in: (a) sampling method – oral rinse versus sub-gingival plaque; oral rinse enables the collection of organisms from the dorsum of the tongue as well as from the ever changing oral mucosal environment as compared to the sub gingival biofilm plaque, (b) detection probe – 18S rDNA probes that detected *Candida* and eight other genera, versus the pan-fungal ITS1/ITS2 probe that could identify all fungi, and (c) sequencing technique – cloning of rDNA fragments followed by sequencing, versus real-time pyrosequencing.

We also found that the distribution of fungal species varied greatly between different individuals. Similar variation was recently reported for bacterial microbiota by Nasidje *et al.* [33], who analyzed the global diversity of the salivary microbiome in 120 healthy individuals and showed that it varied greatly within and between individuals. Our results from the PCO and UniFrac analyses showed some tendency for white males to cluster together, and Asian males to cluster together. However, given the small number of study participants in the current study, it is difficult to draw definite conclusions regarding association of the biome with gender and/or ethnicity. To our knowledge, this is the first study suggesting such trend, and needs to be confirmed in studies involving larger population sizes.

The oral mycobiome of at least 20% of the enrolled individuals included the four most common pathogenic fungi – *Candida* (present in 75% of the cohort), *Aspergillus* (35%), *Fusarium* (30%), and *Cryptococcus* (20%). While the abundance of *Candida* in these healthy individuals was not surprising, the actual percentage was higher than reported in earlier culture-based studies, where 40 to 50% of healthy individuals have been shown to contain *Candida* species in their oral cavity [35],[36]. The high percentage of *Candida* detected in this study could be attributed to the use of the more sensitive ITS/pyrosequencing approach. Another interesting finding was the different types *Candida* species identified in the oral cavity of healthy individuals. The most abundant *Candida* species in this study was found to be *albicans* (in 40% of the subjects), followed by *C. parapsilosis* (15%), *C. tropicalis* (15%), *C. khmerensis* (5%) and *C. metapsilosis* (5%). These results are in agreement with those reported by earlier studies using culture-based as well as PCR-based analyses [35],[36].

The presence of *Aspergillus*, *Fusarium*, and *Cryptococcus* isolates in the oral cavity of healthy individuals was unexpected, since these fungi have not been reported to be colonizers of the oral cavity. It is possible that the pathogenicity of these fungi is controlled in healthy individuals by other fungi in the oral mycobiome, as well as a functional immune system. It is possible that inter-dependent relationships may exist between components of the oral mycobiome, and need to be investigated using broader sampling and longitudinal studies.

In our studies, we also identified 60 fungal genera that are ubiquitous in the environment (present in plants, soil, air) and not normally associated with infections. Among these genera, 39 genera occurred once among the 20 samples analyzed, 16 genera occurred with a frequency of 2, while 5 genera occurred in three individuals. Due to their ubiquitous nature, the presence of these organisms in the oral cavities of healthy individuals was not surprising, which are most likely of environmental origin, from food and mouth breathing.

In this study, non-culturable fungi represented a large percentage (36.1%) of the organisms identified in the oral mycobiome of healthy individuals. This is the first study demonstrating the presence of non-culturable fungal organisms in the oral cavity, which may play important role in the oral milieu. The presence of non-culturable organisms has been reported for bacterial species in the oral cavity; about half the population of oral bacteria has been reported to be non-culturable. For example, Aas *et al.* [4] reported that of the 109 bacterial species identified in the oral cavity of HIV-infected patients, 60% were non-culturable. Although some studies suggested that non-culturable bacteria may be associated with oral disease and health [37],[38], inability to grow these non-culturable organisms renders it difficult to gain insight into their role in health and disease. Moreover, non-culturable organisms may exhibit antimicrobial resistance, which may be the underlying reason for failure to manage certain infections. The field of molecular ecology abounds with examples where molecular methods identify numerous taxa that play important ecological functions but cannot be grown in the lab. Therefore, it is critical to fully characterize ecological communities like the oral mycobiome to fully understand the functionality of that ecosystem.

Ours is the first study that provides a snapshot of the oral mycobiome in various individuals, and addresses an important component of the Human Microbiome Project (HMP). Since this is the first study that identifies the oral mycobiota, our findings complement the HMP (focusing mainly on the bacterial component) and support its stated goal of “generating resources enabling comprehensive characterization of the human microbiota and analysis of its role in human health and disease (http://nihroadmap.nih.gov/hmp/)”. Results from our study provides critical information that is likely to form the basis of further “hypothesis-driven” studies evaluating the oral mycobiome in terms of individual variabilities, longitudinal trends, and the effect of diet and geography, and studies focused on determining the association of oral mycobiota with health and disease.

The clinical relevance for the presence of a diverse population of fungal species in the oral cavity is unknown. It is possible that the presence of a given fungal isolate (e.g. *Candida*, *Aspergillus*, *Cryptococcus*, and *Fusarium*) in an individual could be the first step in predisposing the host to opportunistic infections. In this regard, oral *Candida* colonization has been known to be a risk factor for *Candida* infections in immunocompromised patients [39],[40]. Understanding the relationships between different fungal species as well as between fungi and other members of the oral microbiome will shed light on the pathogenicity of these organisms and may lead to the discovery of novel therapeutic approaches for the prevention and treatment of oral complications.

## Supporting Information

Table S1 Sequence data
(1.15 MB ZIP)

Table S2 Frequency distribution of fungi belonging to different genera present with at least one percent abundance in the oral mycobiome of healthy individuals
(0.05 MB XLS)

Table S3 Species distribution of fungi present in oral cavity of healthy individuals
(0.05 MB XLS)
